# Tegument Assembly and Secondary Envelopment of Alphaherpesviruses

**DOI:** 10.3390/v7092861

**Published:** 2015-09-18

**Authors:** Danielle J. Owen, Colin M. Crump, Stephen C. Graham

**Affiliations:** Department of Pathology, University of Cambridge, Tennis Court Road, Cambridge CB2 1QP, UK; do302@cam.ac.uk (D.J.O.); cmc56@cam.ac.uk (C.M.C.)

**Keywords:** virus egress, virus maturation, herpes simplex virus, HSV-1, pseudorabies virus, PrV

## Abstract

Alphaherpesviruses like herpes simplex virus are large DNA viruses characterized by their ability to establish lifelong latent infection in neurons. As for all herpesviruses, alphaherpesvirus virions contain a protein-rich layer called “tegument” that links the DNA-containing capsid to the glycoprotein-studded membrane envelope. Tegument proteins mediate a diverse range of functions during the virus lifecycle, including modulation of the host-cell environment immediately after entry, transport of virus capsids to the nucleus during infection, and wrapping of cytoplasmic capsids with membranes (secondary envelopment) during virion assembly. Eleven tegument proteins that are conserved across alphaherpesviruses have been implicated in the formation of the tegument layer or in secondary envelopment. Tegument is assembled via a dense network of interactions between tegument proteins, with the redundancy of these interactions making it challenging to determine the precise function of any specific tegument protein. However, recent studies have made great headway in defining the interactions between tegument proteins, conserved across alphaherpesviruses, which facilitate tegument assembly and secondary envelopment. We summarize these recent advances and review what remains to be learned about the molecular interactions required to assemble mature alphaherpesvirus virions following the release of capsids from infected cell nuclei.

## 1. Introduction

The herpesviruses are classified into three subfamilies, the alpha-, beta- and gammaherpesviruses, all of which share a common virion morphology and a group of approximately 40 conserved genes that play key roles during virus replication [1]. Molecular phylogenetic analysis suggests that the *Herpesviridae* subfamilies diverged from a common ancestor around 400 million years ago and evolution over this time has given rise to at least 135 species [2,3]. The herpesviruses occupy a diverse range of biological niches, both in terms of host cell type and length of reproductive cycle. Three alphaherpesviruses are capable of infecting humans: herpes simplex virus (HSV)-1 and HSV-2, which usually cause only mild orofacial or genital lesions, respectively, but can cause more severe disease in neonates or the immunocompromised, and varicella-zoster virus (VZV), the etiological agent of chickenpox and shingles [1]. Pseudorabies virus (PrV), a swine virus that can infect other mammals including monkeys, but not higher primates or man [4], has been extensively studied as a model alphaherpesvirus.

**Figure 1:**
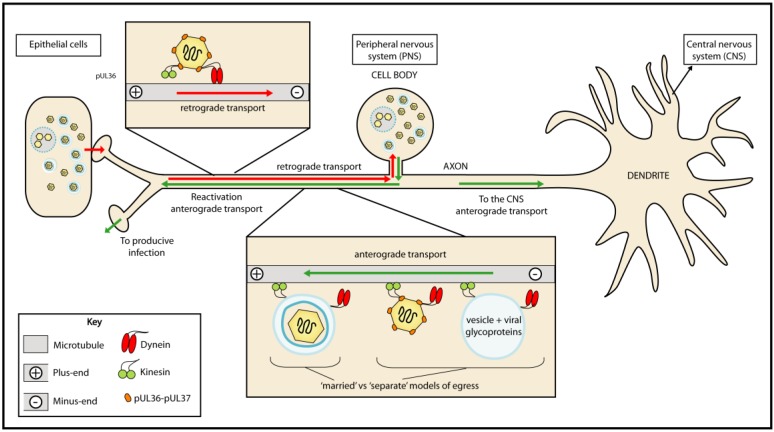
Neuronal trafficking during entry and egress. Alphaherpesviruses establish latent infection in the nuclei of peripheral ganglia following retrograde transport of capsids along microtubules. Reactivation results in the production of new virions that undergo anterograde trafficking back to peripheral tissues. The assembly state of viral particles prior to anterograde axonal transport is disputed and two models have been proposed: the “married model” predicts that virions are assembled in the cell body and trafficked within vesicles; the “separate model” predicts that capsids and secondary-envelopment membranes are trafficked separately with final virion assembly occurring at or near the sites of egress. Minus-end directed transport to the cell body along microtubules is driven by dynein while kinesins drive plus-end directed transport to the cell periphery. The movement of viral particles along axons during entry and egress is bidirectional and saltatory suggesting that both dynein and kinesin motor proteins may be involved. How the net direction of transport during entry and egress is determined is currently unknown.

Alphaherpesviruses are commonly defined by their ability to establish a latent infection in neurons. Primary alphaherpesvirus infection occurs in epithelial cells and is frequently asymptomatic. A lifelong infection is established when virions spread to adjacent sensory neurons, resulting in retrograde transport of capsids to the cell body during viral entry (Figure 1). HSV-1 infects mucosal epithelial cells and latency is established in the maxillary branch of the trigeminal ganglion, part of the peripheral nervous system [5]. During latency the viral genome is maintained in the cell nucleus; periodic reactivation of the lytic cycle leads to the assembly of new viral particles and their anterograde transport along axons to epithelial cells for symptomatic and asymptomatic shedding [6]. Anterograde trafficking and trans-neuronal spread to the central nervous system (CNS) occurs occasionally and is associated with severe disease [7].

**Figure 2:**
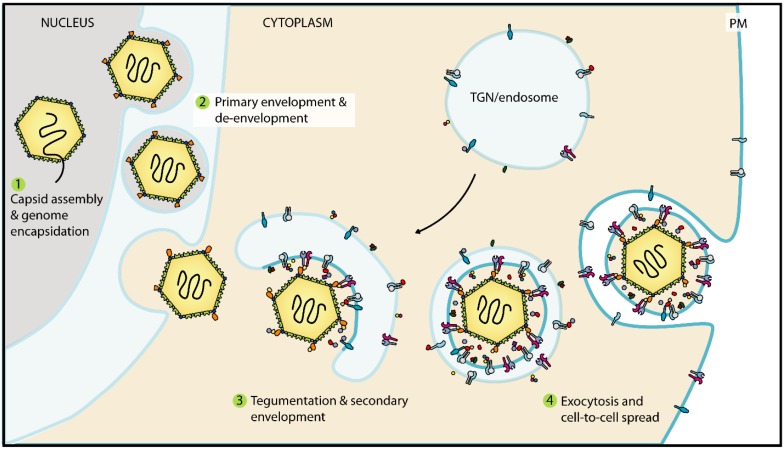
Maturation and egress of herpesviruses. Replication of the viral genome and encapsidation occurs in the nucleus. Once assembled, capsids interact with the inner nuclear membrane and bud into the perinuclear space where they form primary enveloped particles. The primary envelope is then lost upon fusion with the outer nuclear membrane and unenveloped capsids are released into the cytoplasm. Cytoplasmic capsids acquire tegument proteins and their membrane by budding into specialised vesicles, probably derived from endosomes or the trans-Golgi network (TGN), that are studded with viral glycoproteins and outer tegument proteins. The secondary envelopment step also provides a transport vesicle that later fuses with the plasma membrane (PM) to release enveloped virions from the cell.

Herpesvirus virions are organised into four morphologically distinct structures: An electron-dense core that contains the linear double-stranded DNA genome; an icosahedral capsid approximately 125 nm in diameter with *T* = 16 symmetry and a single unique (portal) vertex through which DNA enters and leaves the capsid; a proteinaceous layer termed tegument that links the capsid to the viral envelope; and an outer envelope consisting of host-cell derived lipids, viral envelope proteins and membrane proteins from the host cell [8,9,10,11]. According to the most widely-accepted model, maturation and egress of herpes virions can be described in four stages: (1) capsid assembly and genome packaging in the nucleus; (2) primary envelopment and de-envelopment at the nuclear envelope; (3) tegumentation and secondary envelopment in the cytoplasm; and (4) exocytosis at the plasma membrane or cell-to-cell spread at cell junctions (Figure 2) [12,13,14]. Viral maturation occurs concomitantly with transport through the cytoplasm, as DNA-loaded capsids (so-called “C-capsids”) undergo tegumentation and secondary envelopment *en route* to the plasma membrane. The focus of this review will be on the latter cytoplasmic stages of maturation, tegumentation and secondary envelopment; for reviews of nuclear egress see [12,13,15].

## 2. Tegument Form and Function: An Overview

### 2.1. Tegument Is a Dense Protein Network

Herpesvirus tegument is a self-supporting structure comprising thousands of densely-packaged protein molecules. Proteomic analysis of extracellular HSV-1 virions by mass spectrometry identified 23 virally-encoded tegument proteins and a number of host-cell enzymes, chaperones and structural proteins, some of which are likely to be incorporated into the tegument layer [16]. HSV-1 encoded tegument proteins range in size and abundance, with the smallest predicted to be approximately 10.5 kDa (pUL11) and the largest greater than 330 kDa (pUL36/VP1-2). The most abundant tegument proteins are pUL47/VP13-14, pUL48/VP16, and pUL49/VP22, which are present at 600–1300 copies per virion [17]. Among the host cell proteins identified in extracellular virions are proteins involved in trafficking and exocytosis, in particular members of the Annexin and Rab GTPase families [16]. A myriad of protein:protein interactions bind tegument proteins together so tightly that the structural integrity of this layer is largely maintained even following removal of the membrane envelope from HSV “L-particles”, non-infectious capsid-less particles that are produced during alphaherpesvirus infection [18,19].

### 2.2. Tegument Asymmetrically Links the Capsid to the Viral Envelope

Tegument proteins are generally designated as belonging to either “inner” or “outer” tegument depending on their preferential association with either the capsid or viral membranes during entry and egress, and based on their fractionation behaviour after virion lysis with non-ionic detergents [13,20,21]. More recently the localization of specific tegument proteins within the tegument layer of individual virions has begun to be uncovered using super-resolution microscopy techniques [22]. While the outer HSV-1 tegument appears to be amorphous, the inner layer has partial icosahedral order due to its close association with capsids [23,24,25].

Cryo-electron tomography shows the HSV-1 capsid to be asymmetrically placed inside the viral envelope, with the tegument layer ranging in thickness from approximately 5 nm at the “proximal” pole to around 35 nm at the “distal” pole [23]. HSV-1 particle asymmetry has also been observed by super-resolution microscopy [22] and single-particle fluorescence imaging techniques have shown an asymmetric arrangement of surface glycoproteins and selected tegument proteins around the capsid of PrV virions [26]. This asymmetry is reflected in the distribution of glycoproteins on the outer membrane, with dense clusters of glycoproteins located at the thicker distal pole and relatively few glycoproteins at the proximal pole [23]. Partitioning of glycoproteins in this manner may be consistent with a lack of tegument-based anchorage at the proximal pole and/or the clustering of glycoproteins into lipid rafts. One hypothesis suggests that virion asymmetry may be established during secondary envelopment, with budding initiated at the tegument- and glycoprotein-rich distal pole [23,27]. During cell entry the proximal pole appears to preferentially form the fusion pore with the plasma membrane [27]. Taken together these results suggest that the two poles of the asymmetric HSV-1 virion are functionally distinct: the glycoprotein-rich distal pole mediates assembly while membrane fusion occurs predominantly at the glycoprotein-poor proximal pole [27]. It was proposed that reduced steric hindrance of entry-associated glycoproteins at the less-crowded proximal pole may give rise to an increased frequency of fusion at this pole [27], but this hypothesis has yet to be tested directly.

The icosahedral capsids of herpesviruses have a single unique vertex, termed the portal vertex, that allows DNA packaging during virion assembly and is formed by a dodecameric ring of pUL6 (HSV and PrV) or ORF54 (VZV) [9,28]. A tegument-spanning feature that projects from the portal vertex into the vicinity of the viral envelope and glycoprotein tails has recently been reported for HSV-1 [29]. Designated as the portal vertex associated tegument (PVAT), this feature appears to maintain a constant distance between the portal vertex and the viral membrane, meaning that for virions with a large diameter (*i.e.*, those packaging a large amount of tegument) the portal vertex typically corresponds with the proximal pole. It is unclear whether the orientation of the capsid portal vertex with respect to the membrane helps establish virion asymmetry during secondary envelopment.

### 2.3. Tegument Proteins Modulate the Host Cell Immediately Following Infection

Aside from contributing to the structural integrity of virions, tegument proteins can perform a host of functions within the cell immediately after entry and prior to the *de novo* synthesis of viral proteins. For example, transcriptional activation of immediate-early genes follows the formation of a transcription complex between the host cell factors HCF-1 and Oct-1 and the HSV-1 tegument protein pUL48/VP16 or its VZV homologue ORF10 [30,31,32,33,34]. In HSV-1 and PrV a second tegument protein, pUL41/vhs, suppresses protein expression by degrading host mRNA during the early stages of infection and viral mRNA later in infection [35,36,37,38]. Interestingly, the VZV homologue of pUL41 (ORF17) is not packaged into virions and doesn’t mediate host gene shutoff [39,40].

Infected cell protein 0 (ICP0) is an immediate-early protein that modulates the host’s innate and intrinsic responses to infection (reviewed in [41,42]). The RING domain of ICP0 possesses E3 ubiquitin ligase activity and ICP0 targets several cellular and viral proteins for proteasome-mediated degradation. For example, ICP0 directs the degradation of DNA-PKcs [43], part of the cellular DNA damage response that acts as a cellular sensor of DNA virus infection and potentiates interferon production [44]. ICP0 also disrupts nuclear domain 10 (ND10), also known as nuclear bodies or promyelocytic leukemia protein (PML) bodies, and in doing so disrupts their ability to restrict viral gene expression [41]. ORF61 of VZV is a functional homologue of ICP0 and is able to complement a HSV-1 ICP0 deletion mutant in tissue culture [45]. Similarly, growth defects of the PrV EP0 deletion mutant can be complemented by cells expressing the VZV or HSV-1 ICP0 homologues [46]. ICP0 is incorporated into tegument via its interaction with pUL49 [47], although the exact contribution of *de novo* synthesised *versus* tegument-delivered ICP0 protein in newly infected cells remains to be elucidated [41].

### 2.4. Tegument helps Deliver viral Genomes to the Nuclei of Infected Cells

In addition to modulating the immune system of newly infected cells, tegument proteins direct microtubule-mediated retrograde transport of capsids to the nucleus (reviewed in [48]) and nuclear entry of the viral DNA genome. During entry, HSV-1 and PrV capsids undergo bidirectional and saltatory movement along microtubules with the net retrograde motility directed towards the nucleus [49,50,51,52,53,54]. Bidirectional transport along microtubules suggests that both dynein and kinesin motor proteins may be recruited to incoming capsids [51,55]. Furthermore, dynein and its cofactor dynactin, kinesin-1 and kinesin-2 co-purify with partially tegumented HSV-1 capsids extracted from extracellular virions following incubation with cytosolic extracts [20]. The most likely candidates for recruiting motor proteins are pUL36/VP1-2 and pUL37, since they remain bound to capsids during transit and are able to recruit motor proteins *in vitro* [20,54,56,57,58,59,60,61,62]. A role for pUL36 in retrograde trafficking is supported by the observation that the deletion of the C-terminal 167 residues of HSV-1 pUL36 abolishes directed transport towards the nucleus [62]. Recently, an interaction between pUL36 and the dynein/dynactin motor complex (involved in retrograde transport) has been shown to promote the transport of PrV capsids during entry [63]. While pUL37 has been shown to enhance retrograde trafficking in PrV it is not essential for capsid transport during entry [64].

After trafficking to the nucleus capsids dock and release their DNA genome at nuclear pore complexes (NPCs). Efficient binding of purified capsids to NPCs in an *in vitro* assay was shown to be impaired following the removal of inner and outer tegument proteins by trypsinization [65]. Antibodies targeted against pUL36—but not pUL37, pUL19/VP5 or pUL18/VP23—are able to attenuate capsid attachment at NPCs, suggesting that pUL36 is involved during docking [66]. HSV-1 pUL36 is required for the release of viral DNA from capsids into the nucleus, as demonstrated by a temperature-sensitive HSV-1 virus (tsB7) carrying four point mutations in UL36: at a non-permissive temperature capsids accumulated at nuclear pores but failed to release their DNA [67,68]. Further, a study using artificially-induced syncytia formation showed that nascent viral particles produced by a UL36 HSV-1 deletion virus were unable to infect other nuclei within a syncytium [69]. In contrast, deletion of UL37 didn’t prohibit infection of other nuclei [69]. The capsid protein pUL25 interacts with pUL36, the capsid portal vertex protein pUL6 and the nuclear pore complex protein CAN/Nup214 [70]. The interaction between pUL25 and CAN/Nup214 is also thought to stimulate the release of viral DNA from capsids through nuclear pores into the nucleus of newly-infected cells [70].

### 2.5. Tegumentation and Secondary Envelopment Occurs During Virion Maturation

Herpesvirus virions comprise a complex web of protein:protein interactions between capsid, tegument and membrane-associated viral proteins including surface glycoproteins [71,72,73,74]. The assembly of tegument on capsids occurs predominantly in the cytoplasm following nuclear egress. Partial nuclear localization of tegument proteins pUL36/VP1-2, pUL37, pUL41/vhs, pUL47/VP13-14 pUL48/VP16, pUL49/VP22 has been reported in some studies, but not corroborated in others, and it is unclear whether these proteins associate with capsids within the nucleus [13,62,75,76,77,78,79,80,81]. HSV-1 ICP0 and ICP4 have been detected on purified nuclear C-capsids [82], although this finding is contradicted by other studies [20,47,83]. During virion maturation the inner tegument layer can be observed accumulating on cytoplasmic capsids [84]; outer tegument proteins are targeted to secondary envelopment sites, containing trans-Golgi network (TGN) or endosomal markers, by post-translational lipid modifications or by interacting with the cytoplasmic tails of viral glycoproteins [85,86,87].

During secondary envelopment capsids bud into specialized vesicles containing the glycoproteins that will decorate the surface of mature virions. This process simultaneously provides the viral envelope and also packages the virus into vesicles that later fuse at the plasma membrane. Tegument proteins contribute to this process by forming a network of interactions that bridge the capsid and viral membrane by interacting directly with capsid proteins, with other soluble tegument proteins, and with the cytoplasmic tails of viral glycoproteins or membrane-associated tegument proteins. HSV-1 L-particles, which contain most tegument proteins and all glycoproteins but lack capsids, follow similar assembly and egress pathways to infectious HSV-1 particles, providing evidence that tegument and glycoproteins together are sufficient to drive secondary envelopment [88,89,90].

## 3. Conserved Protein:Protein Interactions Mediate Tegument Assembly

**Figure 3:**
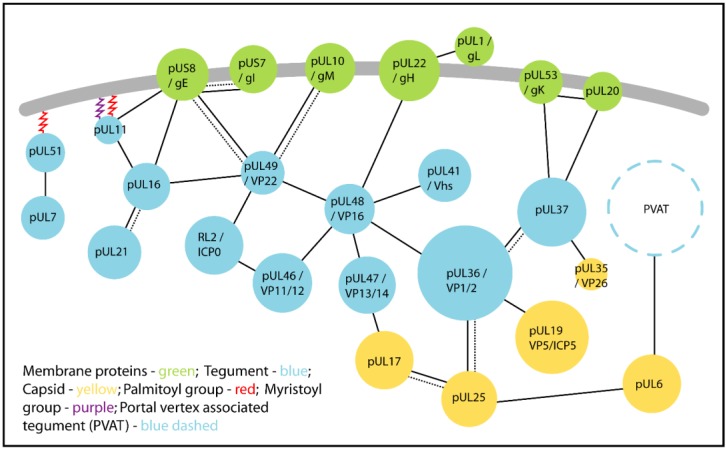
Conserved alphaherpesvirus tegument proteins (blue) link the capsid (yellow) to the glycoproteins and envelope proteins (green) embedded in the virion lipid bilayer envelope (grey). Tegument assembles via a dense network of protein:protein interactions: solid lines indicate interactions demonstrated in HSV and dashed lines show interactions demonstrated for PrV. Some tegument proteins associate directly with the envelope via post-translational modifications conferring lipophilic palmitoyl (red) or myristoyl (purple) groups. The proteins that comprise the portal vertex associated tegument (PVAT) are currently undefined.

Several tegument proteins have been implicated in HSV-1 and PrV secondary envelopment, largely due to the effect of deleting genes encoding these proteins on virus maturation. As shown in Table 1, seven of these genes (UL7, UL11, UL16, UL21, UL36, UL37 and UL51) are conserved in all three herpesvirus subfamilies, while four are unique to the alphaherpesviruses (UL46, UL47, UL48, UL49) [71]. The complex network of protein:protein interactions mediated by its components gives tegument a redundancy that makes characterizing the precise function(s) of any individual protein difficult. In cell culture this redundancy enables the virus to adapt to the deletion of some “non-essential” tegument proteins by increasing the incorporation of other tegument proteins, or of cellular proteins such as actin, into the virion [91,92,93]. While the deletion of many non-essential tegument proteins can often be tolerated there is usually a mild replication defect; a study of PrV shows that the severity of such growth defects in cell culture typically correlates with a proportional increase in mean survival times of infected mice [94]. Despite the challenges, recent studies have begun to elucidate the distinct contributions to secondary envelopment and virus egress made by the tegument proteins conserved across alphaherpesviruses and to map the protein:protein interactions that underpin these functions (illustrated in Figure 3 and summarised in Table 2).

**Table 1:** Herpesvirus tegument genes and their homologues. HSV, herpes simplex virus; VZV, varicella-zoster virus; PrV, pseudorabies virus; HCMV, human cytomegalovirus; EBV, Epstein-Barr virus; KSHV, Kaposi’s sarcoma-associated herpesvirus. Virus subfamily [*Alpha-, Beta- or Gamma-herpesvirinae*] and alternative protein names (in parentheses) are shown.

HSV-1/-2 [*Alpha*]	Mass of HSV Protein, kDa	VZV [*Alpha*]	PrV [*Alpha*]	HCMV [*Beta*]	EBV [*Gamma*]	KSHV [*Gamma*]
*Tegument proteins involved in tegumentation and secondary envelopment*						
UL7	33	ORF53	UL7	UL103	BBRF2	ORF42
UL11	10	ORF49	UL11	UL99	BBLF1	ORF38
UL16	40	ORF44	UL16	UL94	BGLF2	ORF33
UL21	58	ORF38	UL21	UL87	BcRF1	ORF24
UL36 (VP1-2)	336	ORF22 (p22)	UL36	UL48	BPLF1	ORF64
UL37	121	ORF21	UL37	UL47	BOLF1	ORF63
UL51	25	ORF7	UL51	UL71	BSRF1	ORF55
UL46 (VP11-12)	78	ORF12	UL46	-	-	-
UL47 (VP13-14)	74	ORF11	UL47	-	-	-
UL48 (VP16)	54	ORF10	UL48	-	-	-
UL49 (VP22)	32	ORF9	UL49	-	-	-
*Other tegument proteins*						
UL13 (VP18.8)	57	ORF47	UL13 (VP18.8)	UL97	BGLF4	ORF36
UL14	24	ORF46	UL14	UL95	BGLF3	ORF34
UL23 (thymidine kinase)	41	ORF36	TK	-	BXLF2	ORF21
UL41 (vhs)	55	ORF17	UL41	-	-	-
UL50 (dUTPase)	39	ORF8	UL50	-	-	-
UL55	20	ORF3	-	-	-	-
US2	32	-	-	-	-	-
US3	53	ORF66	US3	-	-	-
US10	34	ORF64/69	-	-	-	-
US11	18	-	-	-	-	-
RL1 (ICP34.5)	26	-	-	-	-	-
RL2 (ICP0)	78	ORF61	EP0 (ICP0)	-	-	-
RS1 (ICP4)	133	ORF62/71 (IE62)	IE180 (ICP4)	-	-	-

**Table 2:** Herpesvirus tegument protein interactions that mediate secondary envelopment.

Protein	Other Names	Interaction Partners	Function	References
pUL7	-	pUL51 [HSV-1]	Putative role in cell-to-cell spread and secondary envelopment.	[95]
pUL11	-	pUL16 [HSV-1]	Role in secondary envelopment, also enhances interaction of pUL16 with gE. A tripartite complex of pUL11, pUL16 and pUL21 is proposed to play a role in cell fusion during syncytia formation, possibly through the interaction with gE.	[96,97,98]
		gE [HSV-1]	Cell-to-cell spread and cell fusion during syncytia formation. Glycoprotein E accumulates at the plasma membrane in the presence of pUL11, pUL16 and pUL21, in a cell-type dependent manner. Possible role in secondary envelopment.	[99,100,101]
pUL16	-	pUL11	(See pUL11)	
		pUL21 [HSV-1 and PrV]	pUL21 enhances the interaction between pUL16 and pUL11 in triple-transfected cells. Putative role in cell-to-cell spread, syncitia formation and secondary envelopment when in complex with pUL11 and gE.	[98,101,102]
		gE [HSV-1]	Putative roles in cell-to-cell spread, cell fusion and secondary envelopment. The interaction is enhanced in the presence of pUL11 in transfected cells.	[101,103]
		pUL49 [HSV-1]	Putative role in secondary envelopment.	[104]
pUL21	-	pUL16	(See pUL16)	
pUL36	VP1-2	pUL19/VP5 [HSV-1]	Links the capsid and tegument, essential for tegumentation and secondary envelopment.	[24,25,105]
		pUL25 [HSV-1 and PrV]	Links the capsid and tegument. May be required for stabilisation of the CVSC of nuclear and cytoplasmic capsids. Enhances dynein-mediated transport during PrV entry.	[62,63,70,106,107]
		pUL37 [HSV-1 and PrV]	Provides a scaffold for tegumentation and secondary envelopment. Implicated in enhancing microtubule-based transport during entry and egress.	[74,75,108,109]
		pUL48 [HSV-1]	Contributes to virus assembly. Both proteins are essential in HSV-1 but this is not an essential interaction.	[74,110,111]
pUL37	ICP32	pUL36	(See pUL36)	
		pUL35/VP26 [HSV-1]	Minor role in recruiting pUL37 to capsids.	[72,112]
		gK [HSV-1]	Putative role in secondary envelopment by linking capsid associated pUL37 with the membrane associated complex gK-pUL20.	[113]
		pUL20 [HSV-1]	Putative role in secondary envelopment by linking capsid associated pUL37 with the membrane associated complex gK-pUL20.	[113]
pUL46	VP11-12	pUL48 [HSV-1 and HSV-2]	May regulate pUL48-dependent transcription of immediate-early genes.	[74,114]
		ICP0 [HSV-1]	E3 ligase activity of ICP0 mediates the partial degradation of pUL46 during infection, which may potentiate a shift from immediate-early (α) to early (β) and late (γ) viral gene expression.	[115]
		Many identified in yeast two-hybrid screens	Unknown.	[72,74,116]
pUL47	VP13-14	pUL48 [HSV-1]	Regulation of pUL48-dependent transcription of immediate-early genes.	[110,117]
		pUL17 [HSV-1]	May provide a link between the capsid and tegument.	[118]
		Many identified by yeast two-hybrid screen	Unknown.	[116]
pUL48	VP16/ICP25	pUL36	(See pUL36)	
		pUL41/vhs [HSV-1]	pUL48 inhibits pUL41 during late stage of infection to spare viral mRNAs from degradation by pUL41.	[110,119,120,121,122]
		pUL46	(See pUL46)	
		pUL47	(see pUL47)	
		pUL49 [HSV-1]	Contributes to tegument assembly.	[110,123]
		gH [HSV-1]	May contribute to secondary envelopment.	[124,125,126]
		gD [HSV-1]	Unknown.	[126]
		gB [HSV-1]	Unknown.	[126]
pUL49	VP22	pUL16	(See pUL16)	
		pUL48	(See pUL48)	
		ICP0 [HSV-1]	Packaging of ICP0 into virions.	[47,127]
		gE [HSV-1 and PrV]	Contributes to secondary envelopment.	[99,128,129,130]
		gM [HSV-1 and PrV]	Contributes to secondary envelopment.	[129,130]
pUL51	-	pUL7	(See pUL7)	

### 3.1. pUL36/VP1-2 Interacts with Capsid Protein pUL19/VP5 and Capsid Vertex-Specific Component Proteins pUL17 and pUL25

**Figure 4:**
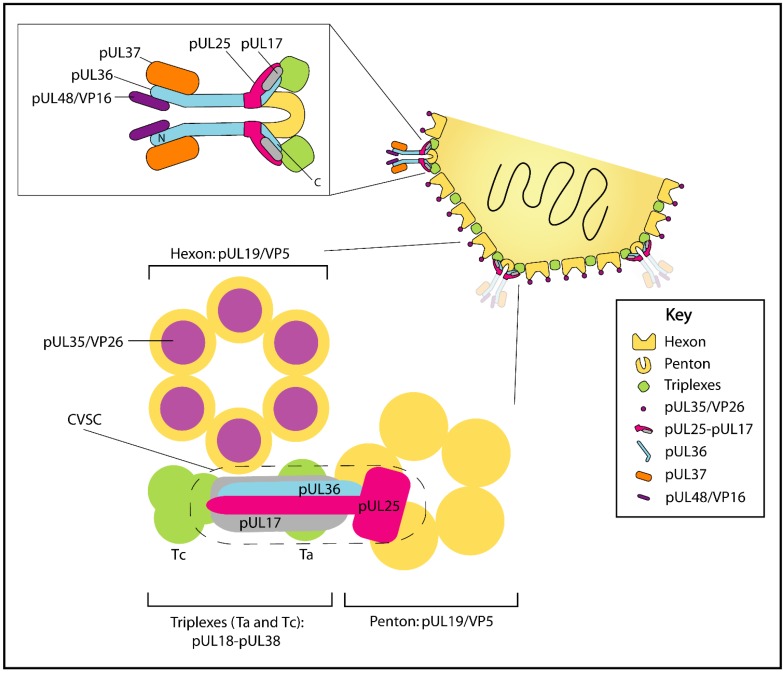
Protein pUL36 extends from capsid vertices and interacts with the capsid vertex-specific component (CVSC). (Top inset) The extended N-terminal region of pUL36 interacts with pUL37 and pUL48. For clarity pUL36 and pUL37 are not drawn to scale. (Bottom inset) Recent studies of HSV, PrV and KSHV [107,135,145] suggest that CVSC component pUL25 lies over the penton vertex, pUL17 lies above the penton proximal pUL18-pUL38 triplex, a C-terminal region of pUL36 contributes to the CVSC density, and that pUL36 is essential for CVSC formation.

The pUL36 protein acts as a foundation stone for tegument assembly by providing a pivotal link between the capsid and tegument structures. The absence of pUL36 from HSV-1 and PrV blocks egress by preventing tegumentation and secondary envelopment, resulting in the accumulation of naked capsids in the cytoplasm [62,131,132]. The major capsid protein of HSV-1 and PrV is pUL19/VP5, which forms pentamers (termed pentons) at the vertices of the icosahedral capsid and hexamers (termed hexons) on the icosahedral faces [10,133,134,135]. Six copies of pUL35/VP26 bind pUL19/VP5 to form a cap that sits over the hexons, and between each penton and hexon are triplexes formed by the proteins pUL38/VP19C and pUL18/VP23 [10,134]. Cryo-electron microscopy (cryo-EM) analysis of C-capsids from mature virions shows pUL36 interacting with the major capsid protein pUL19/VP5 at capsid vertices where it binds between two penton protrusions to form a cap over the penton vertex (Figure 4) [24,25,105]. While addition of pUL36 to capsids had previously been considered to occur in the cytoplasm shortly after nuclear egress, there are now numerous reports of capsid-associated pUL36 being present in the nucleus [75,136,137]. While pUL36 is not essential for nuclear egress of HSV-1 or PrV, a recent study suggested that a nuclear-specific isoform comprising the C-terminal region of pUL36 is recruited to PrV capsids in the nucleus and enhances their nuclear egress [138]. Since the nuclear-specific isoform of pUL36 lacks regions required for virion assembly, it follows that full-length pUL36 can replace the C-terminal fragment at a later stage during maturation [138]. Failure to detect the nuclear localization of pUL36 in some studies but not others may in some cases be explained by the use of antibodies targeting N-terminal epitopes not present in the nuclear-localized C-terminal pUL36 fragment [138].

In addition to binding pUL19/VP5, pUL36 also appears to form part of the capsid vertex-specific component (CVSC, Figure 4). The CVSC is a complex containing the proteins pUL17 and pUL25 that forms over penton-proximal pUL18-pUL38 (VP23-VP19C) triplexes [139,140,141] (Figure 4). Assembly of the CVSC is proposed to promote nuclear egress of DNA-filled C-capsids [140,141,142], although its presence is not exclusive to C-capsids [135,141]. Cryo-EM studies of HSV-1 and PrV capsids have previously positioned pUL17 in the CVSC density closest to the penton vertex and pUL25 in the penton-distal density [135,141,143,144]. However, attempts to fit the crystal structure of a large fragment of HSV-1 pUL25 (47% identity with PrV pUL25) into the CVSC density of PrV C-capsids were unsuccessful and it was proposed that either the folds of HSV-1 and PrV pUL25 are different, or that the conformation adopted by pUL25 differs when in virions *versus* in the crystalline state [135]. Recently, a high-resolution cryo-EM reconstruction of virions showed Karposi’s sarcoma-associated herpesvirus (KSHV), a gammaherpesvirus, to contain a region of tegument near the penton vertices that obeyed icosahedral symmetry, analogous to the CVSC of alphaherpesviruses [145]. However, analysis of this reconstruction in the light of available tertiary and secondary structural data suggested a significantly different arrangement of the pUL17 and pUL25 homologues compared with previous alphaherpesvirus cryo-EM analyses. Specifically, the authors concluded that ORF19, the pUL25 homologue, forms the globular cap over the penton vertex while the penton-distal density is comprised of ORF32, the pUL17 homologue, plus additional density from an unidentified protein. Further, they showed that a similar arrangement of pUL25 and pUL17 would allow a good fit of a dimer of pUL25 into the previously published PrV cryo-EM maps [135,145]. The authors suggest that the elongated nature of the N-terminal segment of ORF19, homologous to a region of pUL25 not present in the crystal structure, would have confounded the interpretation of prior alphaherpesvirus cryo-EM reconstructions performed in the presence of labelled pUL25 [135,141,143] and may have led to incorrect assignment of the pUL25- and pUL17-containing CVSC density [145].

Association of pUL36 with capsids in PrV infected cells is dependent on the expression of pUL25 [106], and homologues of both pUL36 and pUL25 from PrV and HSV-1 co-immunoprecipitate when transiently expressed together in cells [70,106]. In HSV-1, a C-terminal region within pUL36 (residues 2430–2893) is sufficient for recruitment to cytoplasmic capsids during assembly [62]. A recent study of capsids formed by HSV-1 strains lacking UL36 showed that extensive CVSC structures are formed only in the presence of pUL36 [107]. Furthermore, the pUL36 dependence of CVSCs was apparent for C-capsids purified both from the nuclear and cytoplasmic fractions of HSV-1 infected cells [107]. This is consistent with pUL36 (or a C-terminal portion thereof) binding C-capsids in the nucleus, but being replaced with full-length cytoplasmic pUL36 upon C-capsid nuclear exit, and with pUL36 forming part of the CVSC (Figure 4), occupying a position similar to that of the “unidentified” density observed in the KSHV cryo-EM reconstruction [107,145]. We anticipate that higher resolution structural characterization will provide final confirmation of the composition and arrangement of nuclear and cytoplasmic alphaherpesvirus CVSCs and the contribution of pUL36 to these structures.

### 3.2. The pUL36-pUL37 Complex Acts as a Scaffold for Tegumentation and Secondary Envelopment

Protein pUL36 is the largest tegument protein and the N-terminal regions of pUL36 from HSV-1 and PrV interact with the second-largest conserved tegument protein, pUL37 [108]. Protein pUL37 is essential for HSV-1 replication and its deletion severely attenuates replication of PrV [132,146,147]. The pUL37-null phenotype in HSV-1 can be partially rescued by transfecting infected cells with a C-terminal fragment of pUL37 encompassing residues 568–1123 [148]. Recruitment of pUL37 to HSV-1 capsids is dependent on pUL36 [148], and this interaction is conserved between homologues in human cytomegalovirus and KSHV, belonging to the beta- and gammaherpesvirus subfamilies, respectively [149,150,151]. Capsid-bound pUL36 is proposed to extend from capsid vertices and, together with pUL37, to form the filamentous structures that have been observed by cryo-EM of capsids lacking tegument proteins except pUL36 and pUL37 [22,24]. In support of this, a recent crystal structure of a central portion of pUL36 (residues 1600–1733) shows this region to possess an elongated alpha-helical conformation and sedimentation velocity analysis of a fragment encompassing residues 760–1733 is consistent with an elongated structure [152]. A dimer of the pUL36-pUL37 homologues from human cytomegalovirus (pUL48-pUL47) also appears to form a filamentous structure [153].

Unlike most other tegument proteins, pUL36 and pUL37 are incorporated into virions with a fixed stoichiometry and overexpression of pUL37 in infected cells did not increase its incorporation into virions [105,154,155]. The stoichiometry of pUL37 is also maintained in L-particles, suggesting that it is determined by something intrinsic to the tegument structure or viral membrane in the absence of capsids [154]. Interestingly, pUL36 and GFP-tagged pUL37 from HSV-1 have been shown to co-localize with Golgi markers at a juxtanuclear compartment independently of capsids [156]. Precisely how these proteins are recruited to these membranes is unknown, but recruitment of pUL37 depended on pUL36 expression [156]. Direct or indirect interactions with outer tegument proteins, for example pUL36 with pUL48 in HSV-1 [74,111], or with glycoprotein tails may be involved. Recently, immunoprecipitation experiments from infected cells have shown HSV-1 pUL37 to interact with gK and its membrane-associated binding partner pUL20 [113]. The gK-pUL20 complex has been implicated in secondary envelopment and the release of virions from infected cells [157,158,159]. HSV-1 and PrV lacking pUL20, and HSV-1 lacking gK, accumulate enveloped and non-enveloped capsids in the cytoplasm [160,161,162,163,164,165]. It has been proposed that the interaction between pUL37 and the gK-pUL20 complex may contribute to secondary envelopment by linking the pUL36-pUL37 complex with the viral envelope [113]. An interaction between HSV-1 pUL37 and the capsid hexon-cap protein pUL35/VP26 has been detected by yeast two-hybrid screen and may have a minor role in recruiting pUL37 to capsids [72,112].

The exposure of pUL36 and pUL37 on the surface of capsids may facilitate motor protein recruitment for microtubule-based transport of capsids to secondary envelopment sites (reviewed [7,48]). In PrV and HSV-1, both pUL36 and pUL37 have been implicated in microtubule-based transport during egress [57,61,166,167]. Since pUL36 and pUL37 are also linked to retrograde capsid transport during entry there must be an additional factor or condition that determines the overall direction of capsid transport. It is currently unknown how the directional transport of capsids is regulated in cells, but it is likely that a component of the virus particle modulates the switching between dynein and kinesin directed transport [51]. To this end, post-translational modification of pUL36 or the recruitment of other viral tegument proteins to the motor complex have been predicted to play a role [7]. A crystal structure of the N-terminal half of pUL37 from PrV has recently been determined, showing this region to be organized into three domains. One of these domains shares structural similarity with the CATCHR family of multi-subunit tethering complexes [168], complexes that modulate host-cell vesicle trafficking by tethering membranes destined for fusion [169], and the authors postulate that pUL37 may mimic this function and tether PrV capsids to membranes during secondary envelopment.

### 3.3. pUL46, pUL47, pUL48 and pUL49: Central Organizers of Tegument

The pUL46, pUL47, pUL48 and pUL49 proteins act as central organizers of tegument by forming interactions with inner and outer tegument proteins and viral glycoproteins. These are the most abundant tegument proteins in the mature virion and are thought to be unique to the alphaherpesvirus subfamily [16,17,71], although a recent crystal structure of a C-terminal domain of pUL49/VP22 showed unexpected structural homology with ORF52 from murid herpesvirus 4, a gammaherpesvirus [170]. Of the four proteins only pUL48 is considered to be essential for HSV-1 replication in tissue culture [117,127,171,172,173,174]. In PrV, deletions of each of these four proteins individually can be tolerated [93,175,176,177]. Furthermore, simultaneous deletion of UL46, UL47, UL48 and UL49 in PrV does not block virion assembly in tissue culture but virus replication is strongly attenuated [178]. Tolerance to deletion of these proteins, as with many other tegument proteins, possibly arises from the redundancy in protein:protein interactions that form the tegument and through compensatory increases in the incorporation of other tegument proteins. For example, deletion of UL47 or UL49 in PrV leads to a specific increase in the packaging of lower molecular weight isoforms of pUL48 [93], and packaging of HSV-1 pUL46 is enhanced in the absence of pUL47 [173].

Secondary envelopment of HSV-1 capsids is abolished in the absence of pUL48 and is strongly suppressed but not abolished in PrV lacking UL48 [171,172,176]. The pUL48 protein of HSV-1 has been shown to interact with the essential inner tegument protein pUL36 [74,110,111], outer tegument proteins pUL41, pUL46 and pUL49 [74,114,119,123], and the cytoplasmic tail of gH *in vitro* [124,125,126]. A cross-linking experiment also highlighted glycoproteins gB and gD as potential pUL48 binding partners [126]. The requirement for pUL48 in HSV-1 secondary envelopment may be attributed to its ability to form numerous interactions that bridge the capsid and virus envelope. In PrV the deletion of UL48 results in the production of large quantities of L-particles that contain pUL46, pUL47 and pUL49, but not pUL36 and pUL37, suggesting that pUL48 could provide a key link between inner and outer tegument [176]. However, while pUL48 interacts with pUL36 and could thus link membranes and capsids, packaging of pUL48 into HSV-1 virions is not decreased when this interaction is abolished [74,110,111]. 

The pUL49 protein has also been proposed to contribute to secondary envelopment through the formation of a tegument-glycoprotein complex comprising pUL49, gE-gI, gM and ICP0, whereby the C terminus of pUL49 bridges the cytoplasmic tails of gE and gM and the N terminus recruits ICP0 [129]. In HSV-1 infected cells, pUL49 is recruited to TGN membranes though interactions with gE and gM [128,129]; packaging of pUL49 into virions is unaffected by deletion of either glycoprotein individually but a double deletion virus fails to incorporate pUL49 [129]. Glycoprotein I (gI) is recruited to the complex through an interaction with gE; mutants lacking pUL49 incorporate less gE-gI and ICP0 is also absent [127,179]. pUL49 of PrV has also been shown to interact with gE and gM by yeast two-hybrid screen [130]. Triple mutant viruses lacking gE-gI and gD (HSV-1) or gM (PrV) accumulate unenveloped cytoplasmic capsids, suggesting that these proteins play important but redundant roles in secondary envelopment [180,181]. Additionally, a tripartite complex comprising pUL49-pUL48-pUL41 has been proposed, which may promote secondary envelopment through the pUL49-gE-gI complex (reviewed [12]). However, despite the central role of pUL49 in these complexes the deletion of UL49 in both HSV-1 and PrV does not have a significant effect on virus assembly and a PrV UL49 deletion virus does not exhibit an obvious defect in secondary envelopment [93,127,174]. Interestingly, HSV-1 pUL49 has also been shown recently to co-localize with the N-terminal domain (residues 1–155) of pUL16 in the absence of other viral proteins, and pUL49 is very poorly incorporated into pUL16-null virions [104]. However, the contribution this interaction makes to HSV virion maturation is currently unclear. It is also worth noting that deletion of UL49 from HSV-1 causes mutation of UL41 when viruses are propagated in non-complementing cells, presumably due to pUL49 being important for controlling the activity of pUL41/vhs [182,183,184]. Caution may therefore be needed when interpreting the impact of pUL49 removal as, depending on how the UL49 deletion viruses have been propagated, some of the observed phenotypes may actually arise from changes in pUL41/vhs activity.

Little is currently known about the precise contributions of pUL46 and pUL47 to virus assembly. A PrV UL47 deletion mutant accumulates aggregates of partially-tegumented capsids in the cytoplasm and exhibits a 10-fold reduction in viral titer, but no assembly defect was apparent for the UL46 deletion mutant in this study [175]. Mean survival times of mice infected with a PrV UL46 deletion virus were similar to those of the wild-type virus, while survival times increased for mice infected with UL47-, UL11- or UL48-deleted PrV viruses [185]. Yeast two-hybrid interaction screens have identified several capsid (pUL19/VP5, pUL18/VP23, pUL38/VP19C, pUL25), tegument (pUL21, pUL37, pUL48, pUS3, pUS10) and membrane (pUL45, gK, gM) proteins as potential binding partners for pUL46 [72,74,116], suggesting that this protein is able to bridge the capsid to the outer membrane. Interactions between pUL46 and pUL21, pUS3, pUS10 and gM were also identified by mass spectrometry following immunoaffinity purification from cells infected with a HSV-1 GFP-pUL46 virus, as were interactions with ICP0 and a number of host-cell protein kinases [115]. In HSV-2 infected cells pUL46 has been shown to co-localize and co-purify with pUL48 [114] and this interaction has also been confirmed for HSV-1 by *in vitro* GST pull-down experiments [74]. Similarly, pUL47 forms numerous interactions with other tegument proteins (pUL14, pUL21, pUL48, pUL49, pUS11) in yeast two-hybrid interaction screens [116]. Immunoprecipitation of pUL47 with pUL48 from HSV infected cells has been reported [110], and the absence of pUL47 during infection diminished pUL48-mediated reporter gene transcription [117]. The capsid protein pUL17 has also been shown to co-immunoprecipitate and co-localize with pUL47 from HSV-1 infected cells and may provide an additional link between the capsid and tegument [118]. Despite the many pUL46 and pUL47 interaction partners identified by yeast two-hybrid or proteomic studies only a few have been validated by *in vitro* or cell-based experiments. Further investigation is therefore required to ascertain the precise contributions of pUL46 and pUL47 to virus assembly.

### 3.4. pUL11, pUL16 and pUL21 form a Tripartite Complex

In addition to the glycoproteins embedded within the envelope, HSV-1 virions contain several membrane-associated tegument proteins [186]. One such protein, HSV-1 pUL11, associates with Golgi membranes in infected cells via myristoyl and palmitoyl anchors, the latter modification determining membrane specificity [187,188,189]. Partitioning of HSV-2 pUL11 into lipid rafts has been shown to require both acyl modifications [190]. HSV-1 pUL11 membrane association may also be facilitated though an interaction of this protein with the cytoplasmic tail of gE, which has been demonstrated *in vitro* [99,100,101]. Virion packaging of pUL11 is substantially reduced upon deletion of the gE cytoplasmic tail, and a reciprocal defect in gE packaging is also observed upon deletion of UL11 [100]. Although UL11 is not essential for viral replication, deletion of this gene or its homologues from HSV-1, PrV or human cytomegalovirus leads to defective secondary envelopment and an accumulation of cytoplasmic capsids to varying degrees [191,192,193,194,195,196]. Interestingly, HSV-1 pUL11 expressed without acyl modifications partially recovered the growth defects of a UL11 deletion virus, demonstrating that some pUL11 function is maintained when the protein is not membrane-bound [197].

The tegument protein pUL16 has been shown to interact with pUL11 [96,97,98] and gE [101,103] in HSV-1 and with pUL21 in both HSV-1 and PrV [98,101,102]. Both pUL16 and pUL21 are simultaneously pulled-down by GST-pUL11 from lysates of cells infected with wild-type HSV-1, while GST-pUL11 fails to pull-down pUL21 from lysates infected with a UL16 deletion virus [98]. It is also likely that GST-pUL11 of PrV is able to pull-down pUL16 and pUL21 homologues from PrV-infected lysates [98]. Furthermore, packaging of pUL11 and pUL21 is severely reduced in a virus lacking UL16 and the amount of pUL16 incorporated into virions is drastically reduced in the absence of either pUL11 or pUL21 [104,198]. Together these findings provide support for the formation of a tripartite complex with pUL16 at the centre linking pUL11 and pUL21.

**Figure 5:**
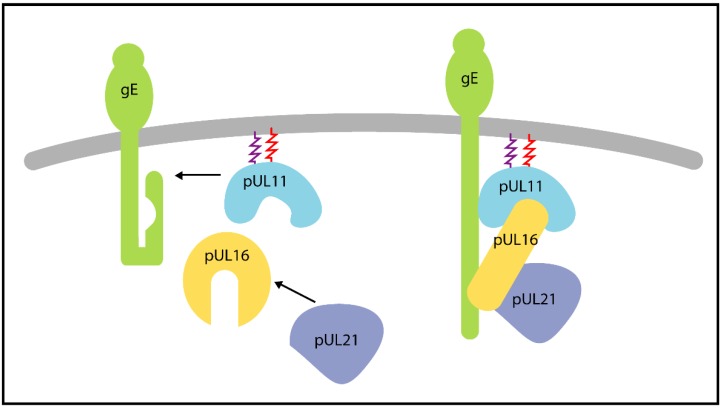
Proteins pUL11, pUL16 and pUL21 may form a tripartite complex that binds gE. The C-terminal domain of pUL16 inhibits its ability to co-localise with pUL11 and gE, co-localization of pUL16 with pUL11 is enhanced in the presence of pUL21, and the presence of pUL11 promotes co-localization of pUL16 and gE [101]. An alternative model is that pUL16 acts as a molecular chaperone, promoting the correct folding of pUL11, pUL21 and/or gE.

GST pull-downs experiments have demonstrated a direct interaction between HSV-1 pUL16 and gE *in vitro* [103]. However, transfection experiments showed that co-localization between pUL16 and co-transfected gE or pUL11 in the absence of virus infection is poor [101,103]. Truncation experiments revealed that the C terminus of pUL16 negatively regulates its interaction with gE and pUL11: removing the C-terminal region (residues 156–373) enhanced the ability of pUL16 to co-localize with pUL11 or gE in co-transfection experiments [103,199]. Co-transfection of pUL16, pUL11 and pUL21 together leads to the efficient co-localization of pUL16 with pUL11, suggesting that pUL21 can relieve the repression of pUL11 binding that is conferred by the pUL16 C-terminal region [101]. Further co-transfection studies show the interaction between pUL16 and gE to be substantially enhanced in the presence of pUL11 but less so in the presence of pUL21 [101]. Therefore, it has been proposed that pUL11-pUL16-pUL21 complex is able to interact with membranes by assembling on the cytoplasmic tail of gE via pUL11 and via pUL16, with direct binding of pUL11 to gE promoting the gE-pUL16 interaction [101] (Figure 5). An alternative hypothesis is that pUL16 acts as a virus-encoded chaperone, promoting the correct folding of pUL11, pUL49 and gE rather than forming molecular interactions to bridge them directly [104,199].

In HSV-1 infected cells pUL16 has been shown to associate with capsids in the cytoplasm independently of pUL36 and pUL37 [198,200]. PrV pUL21 is also reportedly capsid associated [201]. These observations led to the proposal of a simple hypothesis whereby the interaction between membrane-associated pUL11 and capsid-associated pUL16 (and pUL21) could provide a physical link between the capsid and tegument to promote secondary envelopment [12]. If this were the case we might expect a similar phenotype for the deletion of UL16 as is observed upon deletion of UL11. However, these viruses are phenotypically distinct: the HSV-1 UL16 deletion virus forms clusters of membrane-associated capsids in the cytoplasm and has an increased propensity to form multi-capsid virions [104], whereas deletion of UL11 from HSV-1 or PrV causes the cytoplasmic accumulation of non-enveloped capsids in association with electron-dense tegument-derived material [191,192,196,202]. Further experiments are thus required determine how these proteins promote correct virion assembly and probe whether pUL16 forms a stable bridge between pUL11 and pUL21 or acts as a virally-encoded chaperone.

### 3.5. pUL51-pUL7 Complex May Promote Secondary Envelopment

Another membrane-associated tegument protein implicated in secondary envelopment is pUL51. HSV-1 and PrV UL51 mutant viruses exhibit a reduction in viral titer compared to wild type and accumulate non-enveloped capsids in the cytoplasm [203,204]. HSV-1 pUL51 localizes at the Golgi in transfected cells through an N-terminal palmitoyl modification [205]. The localization differs in infected cells, with pUL51 clustering at a juxtanuclear assembly compartment and at the perinuclear compartment [205]. Recently HSV-1 pUL51 has been reported to interact with a second tegument protein, pUL7, which also localizes to a juxtanuclear compartment during infection [95,206]. In infected and transfected cells pUL7, pUL51 and gE partially co-localize on cytoplasmic membranes and this co-localization is lost in the absence of pUL51, suggesting that membrane localization of pUL7 is specifically mediated by the pUL51 protein [95]. Efficient packaging of pUL7 into virions was also shown to be dependent on pUL51 [95]. Furthermore, the phenotype for UL7-null PrV is similar to that described for the pUL51-deficient PrV and HSV-1 viruses [206,207]. The HSV-1 UL7 deletion virus is also defective for cellular egress, but it is unknown whether this is due to impaired secondary envelopment [208]. Taken together these findings suggest that pUL7 and pUL51 function as a complex during viral maturation that promotes secondary envelopment, although how they act in concert to achieve this is currently unclear.

## 4. Secondary Envelopment and Viral Egress

### 4.1. Secondary Envelopment Occurs at Post-Golgi Membranes

There is little doubt that alphaherpesviruses acquire their final lipid envelope from post-Golgi membrane compartments, although the precise identity of the cytoplasmic compartment(s) where secondary envelopment occurs has been the topic of much recent debate. Many studies have provided evidence for the TGN being the site of HSV-1, PrV and VZV secondary envelopment, primarily through analysis of cellular markers of the TGN and treatments that disrupt TGN function [84,209,210]. More recently, evidence for endosomal membranes being the sites of HSV-1 secondary envelopment has been published, where endocytic tracers were localized to sites of secondary envelopment and the early endosome regulator Rab5 was shown to be important for HSV-1 envelopment [86]. Classically the TGN is considered as a major sorting organelle within the secretory pathway that directs newly synthesized proteins, after appropriate processing and transit through the Golgi, to various cellular destinations including endosomes, lysosomes and the plasma membrane [211,212]. However, the TGN also receives proteins from the endocytic pathway, including proteins internalized from the plasma membrane, and so this organelle can also be considered part of the endosomal system [213,214]. Membrane traffic within the secretory and endocytic pathways comprises of a network of highly dynamic and interconnected compartments that rapidly transport proteins between each other using vesicle carriers and/or direct fusion, and so the content of different compartments can mix in various locations. Cellular membrane proteins that are used as markers of the TGN and other post-Golgi compartments predominantly localize to these compartments at steady state, by definition, but often do so in a highly dynamic fashion involving traffic to and from endosomal compartments and the plasma membrane in normal cells. Therefore, data demonstrating the localization of TGN markers and endocytic tracers to HSV-1 secondary envelopment sites are not necessarily contradictory given the intimate association of the TGN with the endocytic pathway. One of the problems faced when attempting to define membrane compartment identity in infected cells is that HSV-1 is known to dramatically re-organize the cytoskeleton and the secretory and endocytic pathways [215,216]. Therefore, assessing the origin of secondary envelopment membranes, which are likely to be quite heterogeneous, is not straightforward. These issues may be clarified in the future by examining the dynamics of HSV-1 envelope protein transport in parallel with cellular “compartment-marker” membrane proteins in infected cells, and by probing the specific involvement of different endocytic and exocytic vesicle transport pathways in HSV-1 secondary envelopment.

### 4.2. Trafficking of Glycoproteins to Sites of Secondary Envelopment

Whatever the precise origin of the membranes that make up the secondary envelopment compartments, all the viral membrane proteins destined to become part of the mature virion must be trafficked to these sites. Given the varying expression kinetics of the 16 different virally encoded membrane proteins that are thought to be in the mature virion (gB, gC, gD, gE, gG, gH, gI, gJ, gK, gM, gN, pUL20, pUL43, pUL45, pUL56 and pUS9), it seems likely that many of these membrane proteins will be trafficked to secondary envelopment compartments either independently, or in subcomplexes, rather than as a fully-formed cluster of all 16 different membrane proteins in the correct stoichiometry. Many of these viral envelope proteins contain consensus sequences within their cytoplasmic domains that would be predicted to mediate their subcellular localization via interactions with cellular protein sorting machinery. This has most clearly been demonstrated for viral membrane proteins such as gB and gE, which contain tyrosine-based motifs that are known to interact with clathrin adaptors and are important for the endocytosis and intracellular localization of these glycoproteins [217,218,219,220]. However, several viral membrane proteins do not contain any predicted trafficking motifs and thus rely on other viral proteins for their correct localization and incorporation into virions. For example, in HSV-1 it appears that localization of the gE-gI heterodimer to the TGN relies on trafficking motifs within the cytoplasmic domain of gE [221], and the ER-exit and TGN localization of gK and pUL20 has been shown to be interdependent [165]. It has also been shown that the essential HSV-1 entry proteins gD and gH-gL rely on the trafficking activity of gM and/or gK-pUL20 to mediate their endocytosis and localization to sites of secondary envelopment [163,222,223]. While the molecular details of how many of the viral envelope proteins are localized to secondary envelopment compartments are unclear, it seems likely that a combination of different endocytic and/or other vesicle transport pathways bring these membrane proteins together in the same location. Presumably, the still poorly-defined network of protein:protein interactions linking envelope proteins with each other and the underlying tegument will then provide sufficient structure and stability to allow the budding/wrapping process to occur, normally around a capsid to form virions, but also occurring in the absence of capsids in the case of L-particles.

### 4.3. The End of Secondary Envelopment: Membrane Scission Mediated by the Host-Cell ESCRT Pathway

The final stage in herpesvirus virion assembly can be considered as the membrane scission event that separates the newly formed virus from the surrounding host-cell membrane, giving rise to a fully formed virion contained within the lumen of a large transport vesicle. Topologically, this is the same process that is required for the final stages of budding for any enveloped virus, whether such a virus buds directly through the plasma membrane (such as HIV) or into the lumen of a cytoplasmic organelle (such as herpesviruses). Many enveloped viruses have been shown to utilize the membrane scission activity of the cellular endosomal sorting complex required for transport (ESCRT) machinery for this crucial step in their assembly [224]. Herpesviruses appear to be no exception: ESCRT function is important for the assembly of members of all three subfamilies of herpesvirures as has been demonstrated for HSV-1 and PrV (alphaherpesviruses) [225,226,227,228], human cytomegalovirus (betaherpesvirus) [229] and Epstein-Barr virus (gammaherpesvirus) [230], although in the case of Epstein-Barr virus the main defect observed in the absence of ESCRT function was in nuclear egress. The ESCRT machinery is a set of multiprotein complexes that are normally involved in the formation of intraluminal vesicles by an inward budding process into the lumen of multivesicular bodies, as well as membrane scission during the abscission event that separates cells at the end of cytokinesis [231,232]. It is important to note that a requirement for the activity of the ESCRT machinery during herpesvirus secondary envelopment does not help define the source of membrane for cytoplasmic assembly compartments. The ESCRT machinery is relatively mobile and can be recruited to multiple cellular membranes under different conditions, and so the involvement of the ESCRT machinery in itself does not allow any firm conclusions to be drawn on sites of HSV-1 envelopment. Currently it is unclear how herpesviruses recruit and regulate the ESCRT machinery at sites of their assembly. By analogy with other viruses that are known to recruit ESCRT complexes via their matrix proteins (see Table S1 in [224]), tegument protein(s) appear the most likely candidates to directly or indirectly interact with ESCRT proteins. Indeed HSV-1 pUL36 has recently been shown to interact with TSG101, a component of the ESCRT-I complex, although the importance of this interaction for secondary envelopment was not investigated in this study [233]. However, given the complexity of the tegument, as well as the redundant nature of protein:protein interactions within the tegument, recruitment of the ESCRT machinery to sites of secondary envelopment seems likely to be mediated by multiple viral proteins.

### 4.4. Trafficking of Assembled Virions to the Plasma Membrane

Currently there is little understanding of how mature herpesvirus virions are released from cells following secondary envelopment. Undoubtedly these viruses utilise proteins involved in the host cell secretory pathway to facilitate egress at the plasma membrane. In particular, Rab3A, Rab6A, Rab8a, Rab11a, GAP-43, kinesin-1 and SNAP-25 have been shown to traffic with viral tegument and glycoproteins to egress sites for HSV-1 and PrV [234,235]. Amyloid precursor protein, an integral membrane protein, co-purifies with intracellular HSV-1 viral particles and has been shown to enhance trafficking of particles with GFP-labelled pUL35/VP26 during egress, possibly by recruiting kinesin-1 [236]. The cellular membrane traffic mediators protein kinase D, which regulates the exit of secretory cargo from the TGN, and myosin Va, which transports vesicles through the cortical actin network, have also been shown to play a role in the egress of HSV-1 [237,238]. Furthermore, knockdown of Rab6A, Rab10, Rab13 and Annexin1 in a genome-wide siRNA screen was detrimental to HSV-1 replication to varying degrees [239]. Interestingly, knockdown of Rab6A, which is involved in Golgi to plasma membrane transport, inhibits capsid envelopment, further supporting the hypothesis that glycoprotein trafficking to the plasma membrane is a prerequisite for viral maturation [86,240]. Viral glycoproteins and membrane-associated tegument proteins are candidates for recruiting host-cell proteins involved in the secretory pathway since they may be present on the surface of viral transport vesicles. However, as yet there is no understanding of the level of partitioning of viral proteins between the viral envelope and the surrounding transport vesicle membrane.

## 5. Perspectives and Open Questions

As detailed above, recent advances in the field of alphaherpesvirus assembly have illuminated many conserved interactions between proteins that mediate tegument assembly and secondary envelopment. However, despite having mapped interactions between tegument proteins, a functional understanding of how these proteins act together to promote virion assembly remains elusive. The role that host-cell binding partners of tegument proteins play in the process of virion assembly also remain enigmatic. Some outstanding questions of particular interest are as follows:

- What is the precise molecular composition of the CVSC and how does it promote nuclear egress of DNA-loaded capsids?
- What is the protein composition of the PVAT and when does it associate with capsids?
- Are tegument sub-complexes like pUL7-pUL51 and pUL11-pUL16-pUL21 pre-formed in infected cells or do these proteins associate only when tegument is condensing on capsids?
- Do some tegument proteins have non-structural roles in tegument assembly, as hypothesised for the putative virus-encoded protein chaperone pUL16?
- How are the viral glycoproteins transported to and organised within the secondary envelopment compartments?
- What is the source/identity of the cellular membrane used for secondary envelopment and are viral/cellular proteins actively partitioned into virions or virion transport vesicles?
- What are the molecular links between tegument and the cellular ESCRT machinery that promote secondary envelopment?
- How is tegument asymmetry generated and what is the role of PVAT in defining such asymmetry?
- How many tegument proteins act to modulate the host-cell environment immediately following virus infection, before the initiation of viral protein expression?

A molecular understanding of the interactions that mediate alphaherpesvirus assembly should prove insightful for the study of all herpesviruses, illuminating conserved, essential molecular interactions that may be targeted in the design of novel therapeutics and expanding our knowledge of host cell post-Golgi membrane trafficking pathways.
